# Successful multigravid pregnancy in a 42-year-old patient on continuous ambulatory peritoneal dialysis and a review of the literature

**DOI:** 10.1186/s12882-017-0540-7

**Published:** 2017-03-29

**Authors:** Thiam Seong Christopher Lim, Malini Shanmuganathan, Irene Wong, Bak Leong Goh

**Affiliations:** 1 Nephrology Unit, Universiti Putra Malaysia, Selangor, Malaysia; 2 Department of Nephology, Hospital Serdang, 43400 Serdang, Malaysia

**Keywords:** Peritoneal dialysis, Pregnancy, Adequacy, Advance maternal age

## Abstract

**Background:**

For peritoneal dialysis patients, the likelihood of conception is low and the probability of getting through the pregnancy successfully is even lower. Almost 60 years after the first reported case of a successful pregnancy in a dialysis patient, many issues concerning pregnancy in dialysis patients remain unresolved. Our patient’s pregnancy is considered high risk as she has end stage renal failure and falls in the category of advance maternal age for pregnancy. We describe here the course of her uneventful pregnancy which we hope will contribute to the overall knowledge and management of pregnancy in elderly patients receiving peritoneal dialysis.

**Case presentation:**

We report a successful elderly multigravid pregnancy, in a patient undergoing continuous ambulatory peritoneal dialysis (CAPD). Her pregnancy was detected early and she was closely managed by the nephrologist and obstetrician. She tolerated the same PD prescription throughout 36 weeks of pregnancy with daily ultrafiltration of 500-1500mls. Her blood pressure remained well controlled without the need of any antihypertensive medication. Her total Kt/V ranged from 1.93 to 2.73. Her blood parameters remained stable and she was electively admitted at 36 weeks for a trans-peritoneal lower segment caesarian section and bilateral tubal ligation.

**Conclusions:**

At the age of 42, our case is the oldest reported successful pregnancy in a patient on peritoneal dialysis. With careful counselling and meticulous follow up, we have shown that woman in the early stage of end stage renal failure can successfully deliver a full term baby without any complications. Therefore, these women should not be discourage from conceiving even if they are in advanced maternal age for pregnancy.

## Background

Women who conceive while on renal replacement therapy (RRT) are at high risk of encountering life threatening maternal and fetal complications. Elderly pregnancy is associated with an increase in obstetric complications such as antepartum haemorrhage, malpresentation, fetal death, low birth weight, preterm delivery and placenta previa. On the other hand, pregnancy while on RRT increases the risk of neonatal death, premature delivery, miscarriage, maternal hypertension and preeclampsia. These complications are much feared by obstetricians and nephrologists. Peritoneal dialysis (PD) has a significant advantage over haemodialysis as far as a successful pregnancy is concerned. Intensive and customised PD prescription as well as preservation of urine output is the mainstay of management. Maintenance of optimal blood pressure and regular antenatal follow up is of utmost importance.

## Case presentation

We present a case of a 42 year-old mother of 4 with end stage renal disease (ESRD) secondary to IgA Nephropathy. She has been on CAPD since April 2015. Her past obstetric history includes 2 prior spontaneous vaginal deliveries and 2 lower segment caesarean sections. Her first pregnancy was in 1997 and her second pregnancy was in 1998. Both antenatal follow ups were uneventful. She underwent uncomplicated spontaneous vaginal deliveries in pregnancies. Her third pregnancy in 2003 was via emergency caesarean section following the obstetric indication of impending birth asphyxia. In the first trimester of her fourth pregnancy in 2008, she was diagnosed with asymptomatic proteinuria and haematuria. Her renal function was noted to be normal. Towards her last trimester, an antihypertensive drug was initiated for maintenance of optimal blood pressure. She delivered a healthy baby via caesarean section at 36 weeks as her blood pressure remained unfavourable. Unfortunately, she never attended outpatient follow up appointments with us for 5 years following her delivery. In 2013, she reappeared with uncontrolled blood pressure and advance renal impairment. She was referred to the nephrologist and a renal biopsy was performed, confirming the diagnosis of IgA nephropathy. She required initiation of renal replacement therapy in 2015.

She was doing well on CAPD, with four 2 L exchanges per day, using 1.5% bags for each exchange. Her residual urine output was good, around 1000 ml per day during the first trimester and decreased to 600 ml during the last trimester. Five months following the initiation of CAPD, she presented to our PD unit with amenorrhoea. She was subsequently confirmed to be in the 7th week of pregnancy. After providing extensive counselling to the patient and her spouse regarding the potential complications associated with ESRD and advance maternal age, they decided to continue with the pregnancy. She attended monthly antenatal clinic reviews during her first trimester followed by fortnightly reviews from the second trimester onwards. Regular fetal surveillance was carried out. Her medication list was scrutinised to ensure that there were no contraindications in pregnancy. Apart from the initiation of 75 mg asprin once per day, her usual medications which were ferrous fumarate 200 mg per day, folic acid 5 mg per day, vitamin b complex one tab once per day, calcium carbonate 1 g three times a day and bumetanide 1 mg twice a day was maintained. She tolerated the same PD prescription throughout 36 weeks of pregnancy with daily ultrafiltration of 500-1500mls. Her blood pressure remained well controlled between 118–134/75–94 mmHg without antihypertensive medication. Her erythropoietin dosage was increased from the baseline dosage of 4000 iu weekly to 8000 iu weekly during her second trimester, with which she was able to maintain stable haemoglobin readings of between 8.8 and 11.5 g/dL. Her total Kt/V was 2.73 (dialysate 1.41, residual 1.32) during the first trimester and this decreased to 1.93 (dialysate 1.28, residual 0.64) during her third trimester. Serum albumin levels remained within the range of 30–36 g/L. As her obstetric course remained uneventful, she was electively admitted at 36 weeks for a trans-peritoneal lower segment caesarian section (LCSC) and bilateral tubal ligation. The PD catheter was not removed but capped up for future used. She delivered a healthy baby girl weighing 2.5 kg with an Apgar score of 7–8. Both mother and baby were well and discharged the day after delivery. We inserted an internal jugular catheter for her and she now undergoes interim haemodialysis (HD) while awaiting the complete healing of her LSCS scar, before resuming PD.

## Discussion

The first successful pregnancy in a dialysis patient was reported 55 years ago by Confortini [1]. However the first sustained pregnancy in peritoneal dialysis was reported 12 years later in a woman who conceived after 2.5 years on continuous peritoneal dialysis. The pregnancy was sustained until 30 weeks following which the patient went through spontaneous labour and delivered of a stillborn infant [2]. Over time, the successful pregnancy rate has improved from 23% reported by the European Dialysis and Transplant association [3] to over 70% reported by a few case series [4, 5]. An evidence based analysis covering pregnant ESRD women on haemodialysis from 2000 to 2008 showed the overall possibility of a pregnancy resulting in living offspring, ranged from 50 to 100%. However report biases are to be expected as any positive data will invariably be submitted for publication by the successful centres [6].

Pregnancy in ESRD women is relatively difficult, as fertility is markedly reduced due to anovulation and amenorrhea. Despite having normal estradiol levels during the follicular phase of the menstrual cycle, the surge of both leutinizing and follicle stimulating hormone does not occur and this is worsened by low progesterone levels in ESRD women on dialysis. Furthermore, hyperprolactineamia is common and seen in over 70% of women on dialysis. Coupled with reduced libido, it is not surprising that the conception rate has been low and reported to be in the region of 0.3–4.1% [7, 8].

In our patient, due to irregularity of menstruation, the diagnosis of pregnancy was at 7th week. This is much earlier as compare to the mean time of diagnosis of 16.5th week [1]. Despite the presence of residual urine output, the usual urine pregnancy test is not helpful in ESRD patients. Serum levels of the beta subunit of Human chorionic gonadotropin (HCG) are found to be mild to moderately elevated in dialysis patients who are not pregnant. This hormone is produced in small amounts by other cells and it is dependent on the kidney for a substantial part of its excretion. Since a once off serum beta HCG level is not reliable in dialysis patients, pregnancy is normally confirmed by the ultrasound imaging. Fetal cardiac activity can be first detected at 5.5–6 weeks and can be missed if imaging is done earlier due to wrong gestational age dating. If a non-viable pregnancy is suspected in a dialysis patient, the diagnosis should be confirmed by measuring the serial beta HCG as it decreases in a non-viable pregnancy. This will prevent termination of a viable pregnancy in situations where the gestational age has been inaccurately calculated. Early detection of pregnancy is crucial for the nephrologist and obstetrician. Medications that are potentially harmful to the fetus, require substitution or dose modification. Preservation of the mother’s residual urine output, maintenance of dialysis adequacy and nutritional concerns must be handled promptly. It must be highlighted that late diagnosis of pregnancy has been shown to increase maternal and fetal complications [9].

Pregnant ESRD woman have higher maternal and fetal morbidity and mortalitiy rates as compared to women with normal renal function [10]. The likelihood of an infant surviving in women who conceive after initiation of dialysis is 50%. Premature rupture of membrane, polyhydramnios, intrauterine growth retardation, preterm birth, preeclampsia/eclampsia, placental abruption, anaemia, haemorrhage, miscarriage and maternal death are amongst the obstetrical nightmares that may occur. For those on haemodialysis, hypertension and anaemia are frequent maternal clinical concerns whereas intrauterine death and preterm infants were the most commonly reported fetal complications.

The risks are further magnified and compounded when the patient falls in the category of advanced age for pregnancy. Obstetric risks associated with adverse outcome in pregnancy for women aged 35 years and above were quantified in a large retrospective data of 385,120 singleton pregnancies analysed in the North West Thames Region, UK [11]. In this study, pregnant women aged 35–40 years old were at risk of gestational diabetes, placenta praevia, breech presentation, operative vaginal delivery, emergency caesarean section, postpartum haemorrhage, premature delivery, small gestational age and still birth. Women aged 40 years and above had higher odds ratio for the same risks. The study confirmed that advanced maternal age is an independent risk factor for adverse outcomes in pregnancy. On a positive note, older women were significantly more likely to breast-feed and this may reflect more positive attitudes towards breast-feeding in older women. These women usually have greater financial security, social stability and age-related attributes such as emotional maturity and wisdom. Unfortunately the study did not analyse the association of chronic kidney disease (CKD) and its adverse outcomes.

The other complications that occurred may have been attributed to the PD therapy itself. Although peritonitis remains a risk, it can still be treated successfully in pregnancy [12].

Despite multiple challenges faced by ESRD women on dialysis, continuous improvement in maternal–fetal care, dialysis efficiency, frequency and supportive therapy provided by nephrologist and obstetrician has paved the way towards achieving previously inaccessible targets.

Both, PD and HD are feasible modes of renal replacement therapy for pregnant CKD patients. While HD enables precise fluid control, it however may lead to marked hemodynamic instability and blood pressure fluctuations that can impair placental blood flow. PD, on the other hand provides a more gentle and continuous mode of dialysis, with less fluctuations in maternal intravascular volumes. According to Okundaye et al., the occurrence of pregnancy is more common in women on HD rather than those on PD (2.4% versus 1.1%). Once conceived however [12], pregnant chronic dialysis women have better hope for a successful pregnancy while on PD. The reasons include a higher residual renal function, more stable metabolic milieu and the absence of intradialytic hypotension that could potentially cause intrauterine growth retardation and fetal death. Nakabayashi conducted a survey in a Japanese dialysis unit where there were 15 pregnancies conceived on dialysis. Fetal survival was associated with the initiation of dialysis of less than 6 years of duration, residual urine production of equal or more than 50 ml per day, a gestational age of at least 33 weeks and birth weight of greater than median of 1782 grams. All patients who underwent more than 9 years of dialysis did not have a surviving infant [13].

Since PD delivery is very much dependant on the PD catheter, uterine enlargement during pregnancy may alter the catheter position and prescribed therapy efficiency. Respectable residual renal function greatly lessens the impact of poor solute and water clearance due to mechanical causes [14]. The continuous removal of toxic waste and the resulting steady blood toxin levels help to maintain a stable intrauterine metabolic milieu, thus reducing the risk of developing polyhydramnios. Although the pathophysiology of polyhydramnios in ESRD patients is unknown, it seems that having a higher urea level before a HD session may induce solute diuresis by the normal fetal kidneys, thus increasing amniotic fluid production. An alternative or additional explanation may be that frequent and relatively rapid removal of solutes during HD in patients with decreased oncotic pressure may shift free water into the amniotic cavity [15]. Once polyhydramnios develope, it increases the risk of premature labor.

It is not an easy feat to get through pregnancy while on HD, as the patient needs to commit to undergo nocturnal dialysis up to 8 h per night or daily 4 h HD sessions. This demanding HD schedule is one of the main reasons for the improvements in outcomes observed in the past decade. Similarly an obstacle faced by PD patients will be of the increase in intra-abdominal volume in the presence of the gravid uterus that may reduce the available peritoneal cavity for an effective and adequate peritoneal exchange. This concern can be minimized by reducing the peritoneal fluid exchange volumes and by performing more frequent exchanges.

There is also an uncertainty regarding the target clearance for pregnant PD patients. It has been suggested that the Kt/V for PD patients should be increased to the range of 2.2–2.4 for better pregnancy outcomes, but this remains to be proven. To achieve this Kt/V target, therapy volume of up to 20 L per day has been recommended [16]. This approach is not practical in our case as besides doubling up the therapy cost incurred for dialysate, it will also require a PD continuous cycler machine. Instead, most nephrologists would choose to treat the patient clinically, monitoring blood parameters and adjusting the PD prescription as needed rather than following the Kt/v number blindly.

Over the last decade, multiple literatures have stressed the importance of residual urine output with regards to successful pregnancy outcomes [15, 17]. Thus it makes sense to encourage ESRD patients to try for pregnancy early in their course of ESRD if they understand the risks involved and yet are longing to have offspring. However the exact amount of residual urine has not been properly addressed before.

As our knowledge and experience in managing pregnant ESRD women has been improving over the last 4 decades, counselling for ESRD women who wish to conceive, should include the success rate, risks to the mother and baby, importance of adherence to dialysis schedule, and long-term results. In fact there is suggestion that the current dissuasive counselling policy will need to be changed in view of the relatively positive outcomes that were attained over last decade [6].

Closely coordinated follow up between the attending nephrologist and obstetrician specialized in high risk pregnancy is crucial. The absence of other co-morbidities, well controlled blood pressure, stable biochemical parameters, especially haemoglobin and respectable residual renal function strongly contributes to successful and uneventful pregnancy in advanced maternal age. Avoidance of vitamin B12, folate and iron deficiency with vitamin D and calcium administration is safe and recommended [17].

## Conclusion

Our experience with this patient shows that the absence of significant co-morbidities and good initial residual renal function promotes an uneventful and successful pregnancy in women on chronic dialysis despite being in her early 40s. Adequate collaboration and support amongst family, doctors, nurses and the patient is of essence in ensuring that the sweet journey of elderly pregnancy is fruitful and memorable.

## Abbreviation

CAPD: Continuous ambulatory peritoneal dialysis
CKD: Chronic kidney disease
ESRD: End stage renal disease
HCG: Human chorionic gonadotropin
HD: Heamodialysis
LCSC: Lower segment caesarian section
PD: Peritoneal dialysis
RRT: Renal replacement therapy
